# Early Nutrition as a Modulator of Neurodevelopment via the Gut Microbiota in Preterm Neonates

**DOI:** 10.3390/children13070870

**Published:** 2026-06-29

**Authors:** Chrysoula Kosmeri, Foteini Balomenou, Fani Ladomenou, Maria Baltogianni, Vasileios Giapros

**Affiliations:** 1 Child Health Department, Faculty of Medicine, University of Ioannina, 45110 Ioannina, Greece; ckosmeri@uoi.gr (C.K.); faniladomenou@uoi.gr (F.L.); 2 Neonatal Intensive Care Unit, Faculty of Medicine, University of Ioannina, 45110 Ioannina, Greece; f.balomenou@uoi.gr (F.B.); mbaltogianni@uoi.gr (M.B.)

**Keywords:** nutrition, preterm neonates, gut microbiota, neurodevelopment

## Abstract

**Highlights:**

**What are the main findings?**

- Preterm birth disrupts gut microbiota and may impair neurodevelopment.
- Early nutrition, especially human milk, supports beneficial microbiota and the brain.

**What are the implications of the main finding?**

- Optimized nutrition may reduce dysbiosis and neurodevelopmental impairment.
- Microbiota-targeted interventions could improve long-term outcomes in preterm infants.

**Abstract:**

Preterm birth is associated with a higher risk of neurodevelopmental adverse outcomes later in life. This increased risk is partly due to interrupted brain development during critical early periods. Early nutrition is a key factor that can influence both brain growth and the development of the gut microbiota, which plays an important role in overall health. Evidence shows that human milk, including fortified human milk, supports the growth of beneficial gut bacteria such as *Bifidobacterium* and *Lactobacillus*. These bacteria help protect the immature intestine, reduce inflammation, and support normal development. In contrast, formula feeding, frequent use of antibiotics, and perinatal stress, common conditions for preterm infants in neonatal intensive care units, can disturb the normal development of the gut microbiota. These factors may lead to dysbiosis, a state in which harmful bacteria become more dominant. Early changes in gut microbiota may affect brain development through the gut–brain axis. This communication system connects the gut and the brain through immune responses, metabolic products, and hormonal pathways. Disruption of this system during early life may have long-term effects on cognitive, motor, and behavioral development. Both clinical and experimental studies suggest that improving early nutrition may support better neurodevelopmental outcomes. Nutritional strategies such as optimizing protein and energy intake and using probiotics or prebiotics may help promote a healthier gut microbiota and support brain development. In conclusion, early nutrition in preterm infants plays a crucial role during a sensitive period of brain development. By supporting healthy gut microbiota formation, early nutritional interventions may help improve long-term neurodevelopmental outcomes in this vulnerable population. The aim of this narrative review is to explore the relationship between early nutrition, gut microbiota development, and later neurodevelopment in preterm neonates.

## 1. Introduction

An estimated 13.4 million babies were born preterm in 2020 (before 37 completed weeks of gestation), 9.9% of births worldwide [1]. Importantly, complications related to prematurity continue to represent the leading cause of mortality among children under five years of age [2,3]. Although advances in neonatal intensive care have significantly improved survival rates, particularly in high-resource settings, preterm infants remain at substantially higher risk of both short- and long-term morbidity compared to their term-born counterparts. Notably, the severity of adverse outcomes is closely linked to the degree of prematurity [4].

Among the most concerning consequences of preterm birth are its effects on neurodevelopment. Given that neurodevelopmental impairment remains one of the most frequent and life-long sequelae of prematurity, understanding modifiable factors influencing brain development is of particular clinical importance. Even with optimal clinical care, long-term outcomes in cognition, behavior, and mental health remain suboptimal [5]. Preterm infants exhibit an increased risk of neurodevelopmental disorders, including autism spectrum disorder, attention-deficit/hyperactivity disorder, and mood disturbances, as well as motor and sensory impairments such as cerebral palsy, hearing deficits, and altered pain perception [6,7].

A critical factor underlying these outcomes is the disruption of normal developmental processes during a highly sensitive period. The period between 24 and 40 weeks of gestation is a crucial window for brain growth and maturation. In preterm infants, however, this process is abruptly shifted to an extrauterine environment. Most often, this occurs within the Neonatal Intensive Care Unit (NICU) [8,9,10]. This transition exposes the developing brain to conditions very different from the protected intrauterine setting. Preterm neonates are frequently subjected to adverse sensory stimuli, including bright lighting, high noise levels, and repeated painful procedures. They also experience reduced opportunities for positive parental interaction [4,9,10,11]. In addition to environmental stressors, these infants are particularly vulnerable to serious clinical complications such as hypoxic–ischemic injury, intraventricular hemorrhage, sepsis, and bronchopulmonary dysplasia. These conditions can significantly disrupt the normal development of both gray and white matter [4,12]. Consequently, preterm birth is strongly associated with an increased risk of neurodevelopmental impairments [11,13]. Cerebral palsy represents the most severe motor outcome. However, even moderately and late preterm infants often experience long-term difficulties, including attention deficits, language delays, and learning disabilities that may persist into adulthood [14,15,16].

In parallel with brain development, early-life establishment of the gut microbiota has emerged as a key factor influencing neonatal health and development [17,18]. In preterm infants, microbial colonization is profoundly altered. In preterm neonates, the gut microbiota is characterized by delayed maturation, reduced diversity, and dominance of facultative anaerobic and potentially pathogenic taxa, largely reflecting the influence of the NICU environment, antibiotic exposure, and altered feeding practices [19,20]. In contrast to term, breastfed infants, preterm infants exhibit reduced abundance of beneficial anaerobes, particularly *Bifidobacterium* and *Bacteroides*, especially in the early postnatal period [21,22]. Growing evidence suggests that the gut microbiota plays a central role in the bidirectional communication between the gut and the brain, commonly referred to as the gut–brain axis [23,24].

This manuscript aims to explore the role of early nutrition as a key modulator of neurodevelopment in preterm neonates, with a specific focus on its interaction with the gut microbiota. Understanding these complex relationships may provide new opportunities for targeted nutritional and microbiota-based interventions to improve long-term outcomes in this vulnerable population.

A narrative literature search was performed using PubMed, Scopus, and Google Scholar to identify studies published in English up to March 2026. Search terms included combinations of: neonate, preterm infant, prematurity, gut microbiota, microbiota–gut–brain axis, nutrition, human milk, probiotics, prebiotics, and neurodevelopment. Studies were selected based on their relevance to the review topic and scientific quality, with priority given to systematic reviews, randomized controlled trials, and observational studies. The reference lists of the retrieved articles were also screened to identify additional relevant studies that may not have been found in the initial database search. As this was a narrative review, no formal systematic review protocol, predefined inclusion/exclusion criteria, or PRISMA-guided study selection process was applied.

## 2. Composition of Gut Microbiota in Preterm Neonates

The composition of the gut microbiota in early life is highly dynamic, initially characterized by low diversity and gradually evolving into a more complex composition. Initial colonization is dominated by facultative anaerobes, including *Escherichia coli*, *Streptococcus* and *Staphylococcus*, which consume oxygen and thus enable the establishment of obligate anaerobes such as *Bifidobacterium* and *Bacteroides* [17]. As the infant grows and there is a transition from a diet exclusive to milk to the introduction of solid foods, there is a change to the microbiome. Dietary patterns rich in complex carbohydrates tend to promote the expansion of specific fermentative microbial communities, including members of the Firmicutes phylum, whereas diets richer in animal-derived products are associated with increased abundance of *Bacteroides* species. By one year of age, the gut microbiota demonstrates increased abundance of *Akkermansia*, *Veillonella*, *Bacteroides*, and *Clostridium*. The diversity of the gut microbiota enriches and stabilizes into adulthood [25,26].

Gestational age is an important determinant of early microbial development. Compared with term infants, preterm neonates exhibit a distinct gut microbiota characterized by delayed microbial colonization, fewer bacterial species, lower diversity and predominance of potentially pathogenic bacteria [27,28]. Initial microbial communities in preterm infants are typically enriched with low-diversity facultative anaerobes, including members of the Enterobacteriaceae family, as well as *Enterococcus* and *Staphylococcus*, and other hospital-associated pathogens [29]. In term infants, these microbes rapidly consume available oxygen, creating a strictly anaerobic environment that promotes a secondary succession of beneficial obligate anaerobes, including *Bifidobacterium*, *Bacteroides*, and *Clostridium* spp. In preterm infants, this phase is prolonged, resulting in delayed microbial maturation. Although facultative anaerobes are eventually replaced by anaerobic taxa, with increasing diversity by around 30 weeks postmenstrual age, microbial development in preterm infants continues to lag behind that of term peers [30,31]. Additionally, factors such as cesarean delivery and neonatal intensive care unit exposures further disrupt and delay normal microbial succession [32,33].

Overall, the preterm gut microbiome is consistently characterized by reduced colonization with beneficial commensals and an increased abundance of potentially pathogenic bacteria [31,34]. This dysbiotic microbial profile has been linked to a higher risk of adverse clinical outcomes, including necrotizing enterocolitis, altered immune function, and suboptimal neurodevelopmental trajectories. The differences between the composition of gut microbiota in term and preterm infants are shown in Table 1.

## 3. The Impact of Intrauterine Growth on Gut Microbiota

Intrauterine growth may also play a role in the composition of the neonatal microbiota, although available clinical data remain limited. A study has shown that by day 30 of life, SGA preterm infants exhibited lower abundances of *Klebsiella* and *Enterobacter* compared to AGA preterm infants. Moreover, the microbial communities in SGA infants formed distinct clusters, indicating a divergent microbiota profile [35]. Recent evidence further suggests that intrauterine growth restriction (IUGR) is associated with alterations in both placental and meconium microbiomes [23]. Specifically, an increased abundance of the family Neisseriaceae and anaerobic bacteria such as Desulfovibrio has been observed in the placenta of IUGR pregnancies, likely reflecting a hypoxic intrauterine environment [36]. Additionally, IUGR neonates demonstrate a reduced relative abundance of Firmicutes and a lower Firmicutes-to-Bacteroidetes ratio.

This distinct and potentially more pathologic early microbiota in IUGR infants may have important clinical consequences. It is hypothesized that these microbial alterations affect postnatal growth trajectories by modulating the somatotropic axis, including insulin-like growth factor-1 (IGF-1) and growth hormone pathways. They may also influence broader metabolic processes [18]. Consequently, accelerated catch-up growth driven by these mechanisms may predispose preterm IUGR infants to an increased risk of cardio-renal and metabolic complications, such as insulin resistance, obesity, and cardiovascular disease, compared to their AGA counterparts [28].

## 4. Factors Influencing Early Life Gut Microbiota Colonization

Beyond prematurity and intrauterine growth, several other factors can affect early microbiome development. Mode of delivery plays a central role in initial microbial exposure. Infants delivered vaginally are exposed to maternal vaginal and fecal microbiota, promoting early colonization by beneficial anaerobes such as *Lactobacillus*, *Bacteroides*, *Prevotella*, and *Sneathia* [37,38]. These early colonizers rapidly reduce oxygen levels in the gut, promoting the growth of *Bifidobacterium* [39]. In contrast, cesarean section (C-section) bypasses the birth canal, leading to microbial acquisition primarily from maternal skin and the hospital environment. C-section-born infants show reduced diversity, higher Proteobacteria-to-Firmicutes ratios, and delayed colonization by *Bacteroides* and *Bifidobacterium*, with increased presence of *Staphylococcus*, *Corynebacterium*, *Enterococcus*, and *Cutibacterium acnes* [40,41,42]. Although some degree of microbial convergence occurs over time, delivery mode-associated differences may persist beyond the neonatal period. Several studies have reported reduced abundance of *Bacteroides* and delayed microbial maturation in cesarean-born infants for months or even years after birth. However, the persistence and clinical significance of these differences remain heterogeneous across studies and are influenced by factors such as breastfeeding, antibiotic exposure, and environmental conditions [32,33,40].

Postnatal nutrition is another major factor affecting the intestinal gut microbiota during the first year of life [43]. Human breast milk provides commensal bacteria, including Staphylococci, Streptococci, and Bifidobacteria, along with bioactive compounds such as secretory IgA, lactoferrin, and human milk oligosaccharides (HMOs) [44,45,46]. HMOs act as prebiotics, selectively promoting the growth of *Bifidobacterium* and *Lactobacillus* [47]. In contrast, formula feeding is associated with a different microbial profile characterized by greater diversity but a shift toward Firmicutes and Proteobacteria, including *Staphylococcus*, *Klebsiella*, and Enterobacteriaceae [46,48]. Although formula composition has improved, now including bovine milk fat globule membrane and added probiotics like *Bifidobacterium* and *Lactobacillus* species, it does not fully replicate the benefits of human milk [49,50,51]. Infants receiving mixed feeding display an intermediate microbiota profile, with levels of *Bifidobacterium* and *Lactobacillus* between those observed in exclusively breastfed and formula-fed infants [46,52].

NICU-related factors, particularly the widespread use of broad-spectrum antibiotics, significantly disrupt microbial development. Antibiotic exposure reduces diversity, delays beneficial colonization, and promotes overgrowth of Proteobacteria such as *Klebsiella* and Enterobacteriaceae [33,53]. However, antibiotics remain indispensable in the management of suspected or confirmed infections in preterm infants, who are at high risk of sepsis and related complications. Therefore, efforts should focus on optimizing antibiotic stewardship to balance infection control with preservation of microbiota development.

Maternal health also plays a critical role. Maternal obesity and excessive gestational weight gain are associated with increased Bacteroidetes and *Staphylococcus* and decreased *Bifidobacterium* and *Akkermansia* in the infant gut [52,54,55]. Additionally, gestational diabetes is linked to microbiota enriched in *Bacteroides* spp., *Parabacteroides distasonis*, and Enterobacteriaceae, reflecting patterns of dysbiosis [56,57].

Finally, host genetics and environmental exposures within the family influence microbial assembly. The “sibling effect” has been shown to accelerate microbiota maturation, with infants who have older siblings exhibiting higher *Bifidobacterium* abundance and greater overall diversity. In contrast, infants without siblings are more likely to harbor *Clostridium* and facultative anaerobes, with a lower ratio of strict anaerobes to facultative species [42,58].

These factors are summarized in Table 2.

## 5. Mechanisms of the Microbiota–Gut–Brain Axis in Early Neurodevelopment

The intestinal microbiota has emerged as an important regulator of early neurodevelopment through the microbiota–gut–brain axis, a bidirectional communication network linking the gut and the brain. This interaction involves immune, metabolic, endocrine, and neural pathways and contributes to immune maturation, neuroinflammation, and the production of neuroactive compounds. Although the role of the gut microbiota on the infant’s neurodevelopment is not well understood, increasing evidence suggests that the gut microbiota plays a key role in this axis [62].

During the early postnatal period, the developing central nervous system and the gut microbiota undergo rapid structural and functional changes. These processes are closely interconnected through the microbiota–gut–brain axis, a complex bidirectional communication network involving neural, immune, endocrine, and metabolic signaling pathways [63]. Increasing evidence indicates that disruptions in early microbial colonization may influence neurodevelopmental outcomes and contribute to the pathogenesis of neurodevelopmental disorders, including autism spectrum disorder (ASD), attention-deficit/hyperactivity disorder (ADHD), anxiety, and cognitive dysfunction later in life [63].

### 5.1. Immune Modulation and Microglial Maturation

One of the principal mechanisms through which the gut microbiota influences neurodevelopment is immune modulation. Microbial signals are continuously required for the proper maturation and function of microglia, the resident immune cells of the central nervous system [64,65]. During early brain development, microglia are involved in synaptic pruning, shaping neuronal circuits, regulating neural progenitor cells, and maintaining white matter integrity [65,66]. Experimental studies in germ-free animal models have demonstrated that the absence of commensal microbiota results in immature and dysfunctional microglia characterized by altered morphology, impaired immune responses, and a pro-inflammatory phenotype [64,67].

In preterm infants, dysbiosis may further exacerbate neuroinflammation through disruption of intestinal barrier integrity. Increased intestinal permeability (“leaky gut”) facilitates the translocation of microbial products, including lipopolysaccharide and other pathogen-associated molecular patterns, into the systemic circulation [68]. Because the blood–brain barrier in premature neonates is structurally immature, circulating inflammatory mediators and bacterial endotoxins may more readily access the CNS [64,67]. Activation of Toll-like receptor 4 (TLR4) signaling on microglia induces the release of pro-inflammatory cytokines such as interleukin (IL)-1β, IL-6, and tumor necrosis factor-alpha (TNF-α), which contribute to oligodendrocyte injury, impaired myelination, and perinatal white matter injury [46,64,67,69].

### 5.2. Microbial Metabolites and Neuroactive Compounds

Microbial metabolites represent another major pathway linking the gut microbiota to brain development. Short-chain fatty acids (SCFAs), including acetate, propionate, and butyrate, are generated through bacterial fermentation of dietary substrates and exert important neuroprotective and immunomodulatory effects [70]. SCFAs contribute to the maintenance of blood–brain barrier integrity, modulation of neuroinflammation, and promotion of oligodendrocyte differentiation and myelination [46,68]. In addition, butyrate functions as a histone deacetylase inhibitor, thereby influencing epigenetic regulation of gene expression and enhancing the production of neurotrophic factors such as brain-derived neurotrophic factor, which is essential for synaptic plasticity and cognitive development [70,71].

The gut microbiota also regulates the metabolism of dietary amino acids, particularly tryptophan. Microbial metabolism of tryptophan produces indole derivatives capable of crossing the blood–brain barrier and activating aryl hydrocarbon receptors (AhRs) on astrocytes and microglia, leading to suppression of neuroinflammatory pathways [72,73]. Furthermore, tryptophan serves as the precursor for serotonin (5-hydroxytryptamine, 5-HT), a neurotransmitter critically involved in emotional regulation, neurogenesis, synaptogenesis, and axonal development [72,73]. Indigenous gut bacteria significantly influence peripheral serotonin synthesis, thereby indirectly modulating CNS function and behavior [72,73].

Beyond their direct immunomodulatory and epigenetic effects, SCFAs may also act as important mediators linking metabolic and neuroendocrine signaling. SCFAs stimulate enteroendocrine cells to release hormones such as glucagon-like peptide-1 (GLP-1) and peptide YY (PYY) and can activate vagal afferent pathways that transmit signals from the gut to the brain [26,69]. In addition, emerging evidence suggests that SCFAs influence HPA-axis activity by modulating cortisol responses and stress-related neuroendocrine pathways. These observations highlight the integrative role of microbial metabolites in coordinating metabolic, neural, and endocrine communication within the microbiota–gut–brain axis [69,74].

Several bacterial genera have been associated with healthy neurodevelopment during early life. *Bifidobacterium* species are particularly abundant in breastfed infants and contribute to the production of short-chain fatty acids, immune regulation, and maintenance of intestinal barrier integrity [44,47,69]. *Bacteroides* species participate in carbohydrate metabolism and immune maturation [17,25,47], whereas *Lactobacillus* species can produce neuroactive compounds, including gamma-aminobutyric acid (GABA) [48,75,76]. Emerging evidence also suggests a role for *Akkermansia* in maintaining mucosal homeostasis and reducing inflammation [25,55]. Together, these microorganisms may contribute to optimal gut–brain communication through metabolic, immune, and endocrine pathways.

### 5.3. Neural and Endocrine Communication Pathways

Neural signaling through the vagus nerve constitutes another essential component of the microbiota–gut–brain axis. The vagus nerve is the main communication pathway between the enteric nervous system and the brain [75]. The activation of the vagus nerve is partly due to the secretion of chemical signals by endocrine cells in the intestinal tract [76]. Specifically, although many microbial metabolites cannot directly penetrate the blood–brain barrier, they stimulate enteroendocrine cells within the intestinal epithelium to release neuroactive molecules, including cholecystokinin (CCK), GLP-1, PYY, and serotonin [23,75]. These mediators activate vagal afferent fibers and transmit signals to multiple brain regions involved in cognition, emotional regulation, stress responsiveness, and behavior [77].

The gut microbiota also contributes to postnatal programming of the hypothalamic–pituitary–adrenal (HPA) axis, the primary neuroendocrine system regulating the stress response. Germ-free animal studies have demonstrated exaggerated HPA axis activation and elevated cortisol responses following stress exposure, effects that can be partially normalized through colonization with commensal bacteria such as *Bifidobacterium infantis* [78]. Dysregulation of the HPA axis during critical developmental periods may therefore contribute to long-term behavioral and psychiatric vulnerability [77,78].

### 5.4. Clinical Implications for Preterm Neurodevelopment

Disruptions in microbiota–gut–brain signaling during early life are increasingly associated with adverse neurological outcomes in preterm infants. Overgrowth of pathogenic taxa such as *Klebsiella*, *Enterococcus*, and other Proteobacteria has been linked to systemic inflammation, sepsis, and severe brain injury [20,22]. Conversely, a greater abundance of beneficial genera, including *Bifidobacterium* and *Bacteroides*, has been associated with improved cognitive, language, and motor outcomes during early childhood [24,46].

Microbial dysbiosis has also been implicated in several neurodevelopmental disorders. Children with ASD frequently demonstrate reduced abundance of beneficial taxa such as *Bifidobacterium* and *Prevotella*, alongside increased *Clostridia* species, which may promote production of neurotoxic metabolites and elevated propionate levels associated with repetitive behaviors and social impairment [79]. Similarly, altered microbial composition in ADHD has been associated with disturbances in dopamine and serotonin precursor metabolism, supporting the concept that early-life microbiota alterations may influence long-term neurocognitive and behavioral outcomes [80]. It should be noted that the associations observed between specific microbial patterns and neurodevelopmental outcomes do not necessarily imply a causal relationship. Rather, these findings may reflect complex interactions among genetic predisposition, immune maturation, environmental exposures, nutritional factors, and clinical conditions that collectively influence both microbiota development and neurodevelopmental trajectories. Further mechanistic and longitudinal studies are required to clarify the causal role of the gut microbiota in preterm neurodevelopment.

## 6. The Role of Early Nutrition in the Microbiota–Gut–Brain Axis

Nutrition plays a central role in shaping the developing gut microbiota and, consequently, neurodevelopmental outcomes in preterm infants through the microbiota–gut–brain axis [23,59]. The summary of the nutritional strategies in preterm infants that could improve microbiota and long-term outcomes is shown in Table 3. Early feeding practices strongly influence microbial colonization patterns, metabolic signaling, and immune maturation during critical developmental windows [18,20].

Human breast milk is considered the optimal source of nutrition for preterm infants. It provides not only essential nutrients but also bioactive compounds, immunological factors, and commensal microorganisms that contribute to microbiota development [59,60]. Clinical studies consistently demonstrate that preterm infants receiving predominantly mother’s milk exhibit improved neurodevelopmental outcomes, including higher cognitive and language scores, compared with formula-fed infants [59]. These benefits are partly attributed to the human milk microbiome and the vertical transfer of beneficial bacteria, particularly *Bifidobacterium* species, from mother to infant [60,61].

Once established within the intestinal ecosystem, these microorganisms produce metabolites and signaling molecules capable of influencing the CNS through immune, endocrine, and epigenetic mechanisms [23,81]. SCFAs and other microbial metabolites regulate neuronal gene expression, synaptic plasticity, and neuroinflammatory responses, particularly during the first 1000 days of life, a highly sensitive period for neurodevelopment [18,81]. Conversely, dysbiosis in preterm infants has been associated with altered brain organization, impaired cognitive function, behavioral abnormalities, and memory deficits [18,24].

Because preterm infants are especially susceptible to microbial disturbances caused by antibiotic exposure, NICU hospitalization, and formula feeding, optimizing the early nutritional environment represents a promising strategy to support healthy neurodevelopment [20,61]. Interventions targeting the microbiota through human milk feeding, probiotics, prebiotics, and synbiotics may therefore hold significant therapeutic potential for improving neurological outcomes in this vulnerable population [18,59,60,61].

Probiotic supplementation has been widely studied in preterm infants, particularly for the prevention of NEC, late-onset sepsis, and mortality. The most commonly investigated strains include *Lacticaseibacillus rhamnosus* GG and *Bifidobacterium* species (*B. infantis*, *B. breve*, and *B. lactis*). ESPGHAN conditionally recommends *L. rhamnosus* GG or a combination of *B. infantis* BB-02, *B. lactis* BB-12, and *Streptococcus thermophilus* TH-4 to reduce NEC risk, provided strict safety and quality standards are met [82,83]. Although probiotics appear to reduce NEC and improve gut microbial composition, uncertainty remains regarding the optimal strains and treatment protocols. Furthermore, current evidence is insufficient to demonstrate sustained neurodevelopmental benefits, highlighting the need for long-term follow-up studies.

**Table 3:** Nutritional Strategies Targeting the Gut Microbiota in Preterm Infants.

Nutritional Strategy	Effect on Gut Microbiota	Potential Neurodevelopmental Relevance	References
Mother’s own milk	Promotes *Bifidobacterium* and *Lactobacillus*; provides HMOs and immune factors	Associated with improved cognitive and language outcomes	[52,60]
Donor human milk	Provides bioactive compounds, though reduced after pasteurization	Supports gut maturation when mother’s milk is unavailable	[84]
Fortified human milk	Improves protein, energy, and micronutrient intake while preserving benefits of human milk	Supports brain growth and neurodevelopment	[85]
Probiotics	Increase beneficial bacteria, especially *Bifidobacterium* and *Lactobacillus*	May reduce dysbiosis and inflammation	[82]
Prebiotics	Stimulate growth of beneficial bacteria	May enhance SCFA production and immune regulation	[86]
Synbiotics	Combination of probiotics and prebiotics	Potential synergistic effects on gut–brain signaling	[82]
Optimized protein/energy intake	Supports growth and metabolic function	May improve neurodevelopmental outcomes	[87]

## 7. Conclusions

In conclusion, preterm birth disrupts the development of both the brain and the gut microbiota during a critical period of early life. Increasing evidence highlights the important role of the microbiota–gut–brain axis in neurodevelopmental outcomes through immune, metabolic, endocrine, and neural pathways. Preterm infants frequently have gut dysbiosis characterized by delayed microbial maturation, reduced diversity, and increased concentrations of pathogenic taxa, changes that may contribute to neuroinflammation and long-term neurodevelopmental impairment. Early nutrition is one of the most important modifiable factors influencing this process. Human breast milk, through its unique composition of bioactive compounds, commensal microorganisms, and human milk oligosaccharides, promotes the establishment of a more beneficial microbial profile and may support optimal brain maturation. Conversely, factors common in the NICU environment, including antibiotic exposure and formula feeding, can further disrupt microbial homeostasis. Despite growing evidence supporting the role of the microbiota–gut–brain axis in preterm neurodevelopment, several limitations remain. Much of the current evidence originates from animal models or observational human studies, which limits the ability to establish causal relationships. In addition, substantial heterogeneity exists regarding microbiome assessment methods, nutritional interventions, and neurodevelopmental outcome measures. Future research should focus on large multicenter longitudinal studies integrating microbiome profiling, metabolomics, and standardized neurodevelopmental assessments. Well-designed randomized controlled trials are also needed to determine whether microbiota-targeted interventions can produce sustained neurodevelopmental benefits in preterm infants.

## Figures and Tables

**Table 1:** Comparison of Intestinal Microbiota: Term vs. Preterm Newborns.

Feature	Term Newborns	Preterm Newborns
Microbial diversity	Higher	Lower
Dominant bacteria	*Bifidobacterium*, *Bacteroides*	Enterobacteriaceae, *Enterococcus*, *Staphylococcus*
Microbiota maturation	Rapid	Delayed
Beneficial anaerobes	Early abundance	Reduced and delayed
Pathogenic bacteria	Low abundance	Increased abundance
Gut microbiota profile	Balanced	Dysbiotic
Clinical implications	Normal development	Higher risk of NEC and inflammation

**Table 2:** Factors Influencing Gut Microbiota Colonization in Preterm Neonates.

Factor	Effect on Gut Microbiota	Potential Clinical Relevance	References
Prematurity	Delayed microbial maturation, reduced diversity, dominance of facultative anaerobes	Increased risk of dysbiosis, inflammation, NEC, and altered neurodevelopment	[27,29,31]
IUGR/SGA status	Altered placental and gut microbiota; reduced Firmicutes/Bacteroidetes ratio	May affect catch-up growth and long-term metabolic risk	[35,36]
Cesarean delivery	Reduced diversity and delayed *Bacteroides*/*Bifidobacterium* colonization	Delayed immune and microbial maturation	[37,38,41,42]
NICU environment	Exposure to hospital-associated organisms	Increased risk of pathogen colonization and sepsis	[19,20,33]
Antibiotic exposure	Reduced diversity and expansion of Proteobacteria	Greater risk of dysbiosis and NEC	[33,53]
Human milk feeding	Promotes *Bifidobacterium* and *Lactobacillus* growth	Supports immune maturation and neurodevelopment	[43,44,45,46,48,59,60,61]
Formula feeding	Shift toward Firmicutes and Proteobacteria	May contribute to dysbiosis	[46,48]

## Data Availability

No new data were created.

## Abbreviations

The following abbreviations are used in this manuscript:

NICU	Neonatal intensive care unit
IUGR	Intrauterine growth restriction
